# Difference in long‐term care cost obtained with the short‐term intensive prevention service (day service type C): A 3‐year follow‐up study of Japanese older adults

**DOI:** 10.1111/ggi.70102

**Published:** 2025-06-19

**Authors:** Ryota Watanabe, Masashige Saito, Kazushige Ide, Katsunori Kondo

**Affiliations:** ^1^ Center for Well‐being and Society, Nihon Fukushi University Nagoya Japan; ^2^ Center for Preventive Medical Sciences, Chiba University Chiba Japan; ^3^ Faculty of Social Welfare, Nihon Fukushi University Chita‐gun Japan; ^4^ The Japan Agency for Gerontological Evaluation Study Chiba Japan

**Keywords:** home care, preventive care daily life support, reablement, rehabilitation

## Abstract

**Aim:**

A short‐term intensive prevention service, known as day service type C, involves professional intervention for 3 to 6 months to enhance participants' social participation and roles within their communities. This study aimed to evaluate whether implementing short‐term intensive prevention services reduces cumulative long‐term care (LTC) costs over a 3‐year period, compared with the situation for non‐participants.

**Methods:**

This study included older adults aged 65 years and older from Taketa City, Oita Prefecture. A total of 132 individuals participated in short‐term intensive prevention services from 2016 to 2019, and the non‐participant group comprised 116 individuals identified as eligible for services through a self‐administered postal survey in 2019 (both groups at baseline). The non‐participant group was selected as part of the Japan Gerontological Evaluation Study. Both groups were followed up for 3 years from baseline. The cumulative LTC costs derived from the public claims records served as the dependent variable. The covariates were sex, living situation, income, level of long‐term care need, and risk assessment scale. Linear regression analysis was performed.

**Results:**

The participants incurred 241 398 JPY (± 681 335) per person, while the non‐participants incurred 1 147 858 JPY (± 1 244 750). The adjusted linear regression showed that the LTC cost for the participants was lower by 495 534 (−848 382 to −142 686) JPY per person than that for those in the non‐participation group.

**Conclusions:**

Compared with non‐participants, the participants incurred approximately 500 000 JPY less in cumulative LTC costs per person over the subsequent 3 years. The widespread adoption of short‐term, intensive prevention services may contribute to the sustainability of LTC insurance systems. **Geriatr Gerontol Int 2025; 25: 1058–1064**.

## Graphical abstract

A short‐term intensive prevention service reduced cumulative long‐term care costs by approximately 500 000 JPY per participant over 3 years. These findings demonstrate the potential of such services to enhance the sustainability of long‐term care insurance systems.

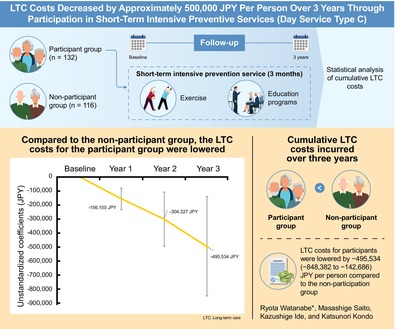

## Introduction

Japan has been experiencing super‐aging ahead of the rest of the world, with the proportion of the population aged ≥65 years reaching 28.9% in 2020.^1^ As aging progresses, the need for social security benefits has also increased.^2^ Long‐term care (LTC) costs are expected to increase from 11 trillion JPY in 2021 to approximately 26 trillion JPY by 2040.^3^ Examining the LTC costs is crucial when considering a sustainable care insurance system.

Since the fiscal year 2015, Japan has progressively implemented Comprehensive Services for LTC Prevention and Daily Life Support, including enhancements in preventive care. This initiative was launched in all municipalities in the fiscal year 2017.^4^ As part of these comprehensive services, a short‐term intensive prevention service, known as day service type C, targets older individuals with impairments in daily living activities. Health, medical, and care professionals engage intensively with these individuals over a short period of 3 to 6 months, to help them regain a fulfilling life, including social participation and finding roles within their communities.^5^ Internationally, such initiatives are referred to as reablement, defined as a holistic, person‐centered, and goal‐oriented approach designed to enhance functionality, increase or maintain independence at home, and reduce the need for continuous services.^6^ In addition, longitudinal studies have shown that among older adults receiving home care in Australia, participants in reablement services incur lower home care service costs compared with non‐participants.^7^, ^8^ Furthermore, although the types of costs differ, reablement participants demonstrate higher cost‐effectiveness in terms of estimated societal costs, including healthcare, home help, and other home‐care‐related expenses, compared with non‐participants.^9^, ^10^ A review that focused on the costs associated with reablement outcomes pointed out that research evidence remains insufficient.^11^ In Japan, randomized controlled trials (RCTs) revealed that individuals who participated in short‐term intensive prevention services had a higher rate of non‐use of LTC insurance services over the subsequent 3 months compared with non‐participants,^12^ suggesting that those using Japan's short‐term intensive prevention services may experience lower LTC costs than non‐participants.

However, the relationship between short‐term intensive prevention services and LTC costs in Asia, including Japan, remains unclear. Owing to the variations in systems across different countries, it is impossible to directly apply the cost findings from previous studies to Japan. Therefore, this study aimed to clarify the relationship between short‐term intensive prevention services in Japan and subsequent LTC costs by comparing participants and non‐participants.

## Methods

This follow‐up study collected data from two groups in Taketa City, Oita Prefecture, Japan, over a 3‐year period: one group participated in short‐term intensive prevention services, while the other did not. Taketa City, with a population of approximately 20 000, had a significantly higher aging rate of 48.2% for those ≥aged 65 years than the rest of Japan in 2022, with Japan's national average being 28.9%. Taketa City was chosen because of its data availability, although Oita Prefecture offers these services to other municipalities.

**Figure 1:**
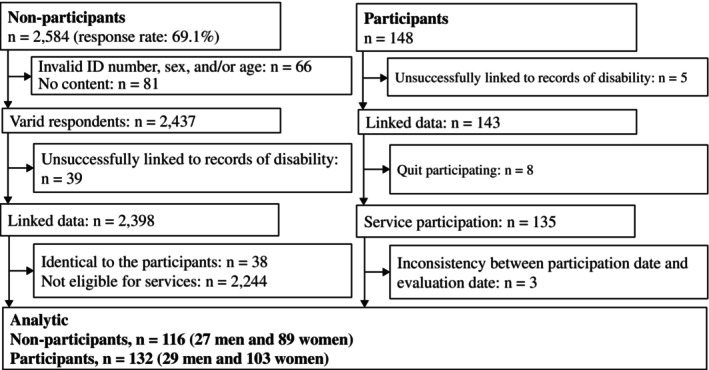
Flowchart of the study population: Participants and non‐participants.

Figure 1 shows a flowchart of the study population. The participant group using short‐term intensive prevention services comprised 132 participants out of 148 individuals who began utilizing these services in Taketa City between the fiscal years 2016 and 2019. Of the 148 individuals, those with unsuccessfully linked records of disability (*n* = 5), those who discontinued use of short‐term intensive prevention services (*n* = 8), and those with errors in their assessment dates (*n* = 3) were excluded, resulting in a final study population of 132. Participants were followed up for 3 years from the start of the service (Fig. S1). Data for non‐participants were obtained from the longitudinal cohort of the Japan Gerontological Evaluation Study (JAGES), a cohort study examining social and behavioral factors associated with health deterioration among adults aged 65 and older.^13^, ^14^ In 2019, the JAGES examined 116 individuals who were selected from 2584 respondents of the Taketa City self‐administered mail survey and followed them up for 3 years (Fig. S1). Exclusions included those with discrepancies in sex, age, or ID (*n* = 66), those without consent (*n* = 81), those unlinked to the long‐term care certification database (*n* = 39), those identified as short‐term intensive prevention service participants (*n* = 38), or independent older adults ineligible for the service (*n* = 2244).

### Dependent variable

The primary outcome of this study was the cumulative LTC cost of public insurance services during the follow‐up period. This LTC cost includes the expenses for “LTC benefits,” “prevention benefits,” and “comprehensive services for LTC prevention and daily life support,” which municipalities report to the National Health Insurance Organizations. In Japan, public LTC benefits services, prevention benefits services and daily life support services under the comprehensive services for LTC prevention and daily life support are available only to individuals who have been certified as requiring support (support level 1 or 2) or care (care levels 1 to 5) or who are experiencing a decline in functional ability based on a checklist.^4^ For non‐participants, the initial assessment point was November 2019, when the questionnaire survey was administered. For the participants, it was the month in which short‐term intensive prevention services commenced, and the cumulative cost of care was then calculated for the subsequent 3 years and 1 month. Japan's LTC system receives services rather than cash benefits and can choose services and providers as needed. Costs were extracted monthly from public LTC claim records maintained by Taketa City. Individuals who died without certification or who did not use services during the follow‐up period were assigned zero cumulative costs. The costs excluded from this study are care management, welfare equipment, home modifications, meal delivery services, monitoring services, some types of day and home‐visit services (with service type C also included in the exclusion criteria), general programs for preventive care, uninsured expenses, such as food and housing, and fully self‐paid services. However, these costs, excluding uninsured coverage costs, accounted for less than approximately 8% of the total LTC costs;^15^ thus they are expected to have a minimal impact on the financial analysis.

The secondary outcomes examined included the duration of LTC service use, non‐utilization rate of LTC services, incidence of disability, and mortality. LTC service duration was determined by the total number of months with incurred LTC costs, and the non‐utilization rate was based on the incidence of LTC costs during follow‐up.^16^ Uncertified individuals were also swept into the non‐underutilization category. The incidence of disability was defined as the incidence of functional disability, which was newly certified as LTCI care level 1 or higher^17^ and LTCI care level 2 or higher,^18^ considering that the study participants were eligible for short‐term intensive prevention services and classified as being eligible for the support level of LTCI. LTCI eligibility was determined based on nationally standardized procedures, physical function assessed by a physician, cognitive function, and assessment by an investigator.^19^

### Independent variable

The independent variable was whether older adults participated in short‐term intensive prevention services. To be eligible for short‐term intensive prevention services, an individual must be assessed as needing support from the LTCI system or be certified as eligible based on the Kihon Checklist.^4^ The participation group was identified from the municipality's list of eligible individuals as those who received short‐term intensive prevention services between the fiscal years 2016 and 2019.

A short‐term intensive prevention service program was offered once a week for 3 months. Before participation, a specialist visited the participants' homes to assess life issues and set goals based on participants’ input. During the study period, a weekly program designed to improve the activities of daily living was offered. Each session lasted 2 h and included stretching, exercises, muscle strengthening training, physical fitness assessments, and lectures on topics related to improving daily living functions, such as dentistry and nutrition. The participants were also provided with written assignments and living records to facilitate their continued efforts at home. Between sessions, they received feedback and consultations from a specialist based on their life records. Moreover, the participants were guided to venues where they could engage in various social activities to continue their social participation after the program ended. After the 3‐month program, a follow‐up visit was conducted to assess whether daily life issues had been resolved and to verify the continuation of social participation.

### Covariates for adjustment

We adjusted for sex, living situation, income category, level of LTC requirement, and risk assessment scale at baseline to align the attributes. Living situations were categorized as alone, couple‐only, or multigenerational households. The income categories were the lowest income, tax‐exempt, and tax liability. The risk assessment scale^20^ included demographic factors, such as sex and age, and 10 questions. Higher scores (0–48) indicate a greater association with disability incidence.^20^, ^21^ Because age is already included as a component of the risk assessment scale (65 years old = 0 points; 90 years and older = 24 points) and is strongly correlated with the total score, it was excluded as a covariate.

### Statistical analysis

The baseline characteristics of the participants are presented using descriptive statistics. Descriptive statistics for primary and secondary outcomes were analyzed and compared.

For the primary outcome, the 3‐year cumulative LTC cost was analyzed using the following methods. First, a classical linear regression model (ordinary least squares, OLS) was employed to control for baseline covariates. In this model, all observations with one or more missing values, which could introduce potential bias, were excluded to facilitate a complete case analysis. Thus, in the main analysis, we performed multiple imputations using multivariate normal imputation to mitigate the potential biases caused by missing values.^22^ Next, we created 20 datasets for all the variables used in the current analysis and subsequently combined the estimated parameters. For the sensitivity analysis, we employed an inverse probability weighting (IPW) estimator to adjust for potential biases.^23^ This method utilizes a dataset that is enhanced using multiple imputation techniques. Furthermore, to observe cost changes over the 3‐year follow‐up period, the analyses were conducted separately for each follow‐up year. Regarding the sensitivity analysis, two types were conducted: (1) an analysis including those who discontinued participation to account for their impact, and (2) a stratified analysis based on eligibility for services and support level to consider baseline care needs.

Secondary outcomes, including duration of LTCI use, non‐utilization rate of LTC services, incidence of disability, and mortality, were analyzed using methods according to each outcome. These methods include linear regression analysis (duration of LTC use), modified Poisson regression analysis (non‐utilization rate of LTC), and Cox proportional hazards analysis (disability and mortality) with multiple imputations.

All statistical analyses were conducted using Stata/MP 17.0 (Stata Corp., College Station, TX, USA), with *P* < 0.05 indicating statistical significance.

## Results

Table 1 shows the participants' baseline demographic characteristics. Compared with non‐participants, those who participated in the service were more likely to be aged <80 years, live alone, have a higher income bracket and a lower LTCI level, and have lower scores on the risk assessment scale.

**Table 1:** Distribution of baseline characteristics in participants and non‐participants.

		Non‐participants (*n* = 116)		Participants (*n* = 132)	
Sex (*n*, %)	Men	27	23.3	29	22.0
	Women	89	76.7	103	78.0
Age (*n*, %)	65–69	3	2.6	4	3.0
	70–74	5	4.3	10	7.6
	75–79	7	6.0	43	32.6
	80–84	25	21.6	45	34.1
	85–89	35	30.2	26	19.7
	90+	41	35.3	4	3.0
Living situation (*n*, %)	Alone	28	24.1	52	39.4
	Couple only	29	25.0	43	32.6
	Multigeneration household	38	32.8	37	28.0
	Missing	21	18.1	0	0.0
Income category (*n*, %)	Lowest income group^†^	56	48.3	36	27.3
	Municipal tax‐exempt	54	46.6	80	60.6
	Municipal tax‐liable	6	5.2	16	12.1
Level of LTC need (*n*, %)	Eligible for services	18	15.5	115	87.1
	Support level 1	42	36.2	11	8.3
	Support level 2	56	48.3	6	4.5
Risk assessment scale (mean, SD)^‡^		32.8	7.3	26.1	7.0

^†^ Based on long‐term care insurance premium.

^‡^ Results for missing are omitted.

LTC, long‐term care; SD, standard deviation.

Table 2 shows the LTCI service data, including cumulative LTC costs during the follow‐up period, status of care required, and incidences of disability and death. Cumulative LTC costs were lower for participants, at 241398 (SD 681335) JPY compared with 1 147 858 (SD 1244750) JPY for non‐participants over 3 years. Similarly, participants exhibited higher rates of LTCI non‐use and lower duration of use, as well as lower incidences of disability and death than non‐participants.

**Table 2:** Descriptive statistics of cumulative cost of LTCI, disability, and mortality during the follow‐up period

					Crude^†^				
	Non‐participants		Participants		B	RR	HR	95% CI	*P*‐value
Cumulative LTC cost (JPY) (mean, SD)	1 147 858	±1 244 750	241 398	±681 135	−906 460	—	—	−1 153 525 to −659 395	<0.001
Duration of use LTCI (months) (mean, SD)	23.8	±14.4	4.8	±9.8	−19.0	—	—	−22.0 to −15.9	<0.001
Non‐use of LTCI (*n*, %)	17	14.7	94.0	71.2	—	4.9	—	3.1 to 7.6	<0.001
Incidence of disability (care Lv1+) (*n*, incidence rate [per 100 person‐years])	56	22.2	32	8.1	—	—	0.36	0.23 to 0.56	<0.001
Incidence of disability (care Lv2+) (*n*, incidence rate [per 100 person‐years])	32	8.0	16	3.9	—	—	0.36	0.20 to 0.65	0.001
Mortality (*n*, incidence rate [per 100 person‐years])	25	6.6	10	2.1	—	—	0.32	0.15 to 0.66	0.002

^†^ To compare results between participants and non‐participants, non‐participants were used as the reference group, and unstandardized coefficients (B), risk ratios (RR), and hazard ratios (HR) were employed for participants. Missing values were imputed using multiple imputations.

CI, confidence interval; LTCI, long‐term care insurance; SD, standard deviation.

For the continuous outcome (duration of LTCI use), unstandardized coefficients were estimated using multiple linear regression. The estimates for the binary outcome (Non‐use of LTCI) were risk ratios estimated by modified Poisson regression. Hazard ratios were calculated for time‐to‐event outcomes (disability and mortality) using Cox regression analysis.

1$ = 150 JPY.

**Figure 2:**
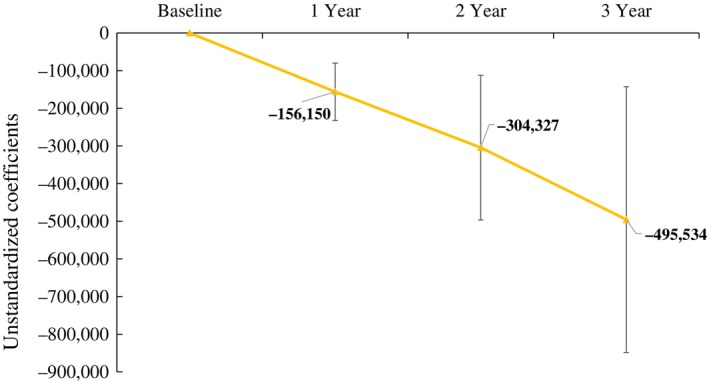
Difference in cumulative LTC costs by tracking period: Comparing participants and non‐participants. Results shown are for the service participants with the non‐participants as the reference.Linear regression analysis was performed using cost as the outcome, with multiple imputation applied to address missing data.Error margins represent 95% confidence intervals.The analysis was adjusted for sex, income, living situation, income category, level of long‐term care need and risk assessment scale.Multiple imputation by chained equations was performed using sex, income, living situation, income category, level of long‐term care need, risk assessment scale, and the cumulative cost of LTCI services (m = 20).The unstandardized coefficient represents cost (JPY).

Table 3 shows the association between the primary outcome and cumulative LTC costs. After adjusting for confounding factors, the cumulative LTC costs for participants, using non‐participants as the reference group, were consistently lower across all models. Specifically, the main analysis using OLS with the multiple imputation model showed a decrease of −495 534 (95% confidence interval: −848 382 to −142 686). Other models also demonstrated reductions of −522 430 (−904 694 to −140 165) in the OLS model and − 516 648 (−784 225 to −249 071) in the IPW with the multiple imputation model. Furthermore, the analysis that included eight individuals who quit the program showed the same trend, although the association was weaker. Similarly, the stratified analysis based on eligibility for services and support level also demonstrated the same trend (Table S1). Figure 2, which shows the relationship between cumulative LTC costs and the follow‐up period, shows that participants' cumulative LTC costs decreased progressively over time.

**Table 3:** Difference in service participants and cumulative cost of LTCI (linear regression analysis)

	OLS			OLS with MI			IPW with MI		
	95% CI			95% CI			95% CI		
	B	lower to upper	*P*‐value	B	lower to upper	*P*‐value	B	lower to upper	*P*‐value
Non‐participants	0.00			0.00			0.00		
Participants	−522 430	−904 694 to −140 165	0.008	−495 534	−848 382 to −142 686	0.006	−516 648	−784 225 to −249 071	<0.001

B, Unstandardized regression coefficient; CI, confidence interval; IPW, inverse probability weighting; MI, multiple imputation; OLS, ordinary least squares regression.

Multiple imputation by chained equations was conducted with 20 imputations (*m* = 20), based on sex, income, living situation, income category, level of long‐term care need, risk assessment scale, and cumulative cost of LTCI services.

OLS and OLS with MI analysis were adjusted for sex, income, living situation, income category, level of long‐term care need, risk assessment scale.

The generalized propensity scores were calculated using multinominal regression analysis using potential confounders: sex, income, living situation, income category, risk assessment scale, and cumulative cost of LTCI services. Additional adjustments were made for level of long‐term care need.

The unstandardized regression coefficient represents cost (JPY).

Regarding secondary outcomes, service participants experienced a shorter duration of LTCI use, a higher rate of non‐use, no difference in the incidence of disability, and a lower incidence of death (Table S2).

## Discussion

The primary finding of this study is that, even after adjusting for baseline factors, participants of short‐term intensive prevention services incurred approximately 500 000 JPY less in LTC costs per person over a 3‐year period compared with non‐participants.

Previous studies have compared home care service costs between participants and non‐participants in reablement services. Results from a longitudinal study demonstrated home care service cost reductions over follow‐up periods ranging from approximately 2 to 5 years.^7^, ^8^ Furthermore, in terms of estimated societal costs, including healthcare, home help, and other home‐care‐related expenses, RCTs and longitudinal studies with follow‐up periods ranging from 6 months to 8 years have shown that participants incur lower costs than non‐participants.^9^, ^10^ The present study supports the above findings. In a study with a 57‐month follow‐up,^8^ home care service costs decreased over time for the participant group, and the present study supports the finding that this difference increased over time. Although the present study followed up for 3 years, it is anticipated that a longer follow‐up period would reveal a greater difference in costs between the participant and non‐participant groups. Including those with incomplete data, the participant group comprised 140 individuals, and it is estimated that the total care costs were reduced by approximately 72 million JPY. This amount is equivalent to 24.6% of the total LTC prevention benefit expenditure for Taketa City from fiscal years 2018 to 2020.^24^ Furthermore, while this study found no statistically significant difference in the prevention of disability progression between participants and non‐participants, differences were observed in the duration of LTC benefit usage, non‐use rate, and mortality rates. This suggests that, although participants may enter a state of LTC need, they use fewer care services and incur lower costs.

Research examining temporal changes in muscle strength training in older adults shows that while a 12‐week program can increase strength, a 6‐week break can reduce these effects by half. However, in this study, long‐term changes were observed despite the short intervention period of just 3 months.^25^ The following three reasons can be considered to explain this outcome. First, the interventions were customized according to the participants' long‐term goals and desired lifestyle, with subsequent lifestyle guidance. According to studies comparing the cost‐effectiveness of reablement through RCTs, incorporating person‐centered goal setting is a primary factor contributing to its effectiveness.^10^ A comprehensive systematic review of person‐centered care indicated that person‐centered care prioritizes proactive and preventive care, enhancing older individuals' ability to perform self‐care by supporting their connections.^26^ Similarly, short‐term intensive prevention services incorporate a person‐centered and goal‐oriented approach. Consequently, these services may have improved the self‐care abilities of participants, enabling them to manage certain needs independently, even in a state requiring care, without reliance on LTCI. The second reason is the support for social participation after the short‐term intensive preventive services. In the present study, after the conclusion of the program, participants were encouraged by their care managers to engage in social participation, and over 80% continued to participate. Although there are no individual records of where each participant connected for social participation, a notable portion of participants attended one of the community gathering places in the city with transportation services for social participation. This location brought together graduates of the short‐term intensive preventive services, potentially fostering the development of social capital. Research targeting community‐dwelling older individuals shows that those who participate in social activities incur lower LTC costs than those who do not,^16^ and the present study supports this finding. The third reason is the development of diverse venues for social participation. Oita Prefecture, the target area of this study, has the highest rate of participation in community gathering places in Japan, and Taketa City has the highest participation rate within the prefecture.^27^ Therefore, participants are in a position to choose from various community gathering places after their completion of the short‐term intensive preventive service program. This implies that, considering these three factors, the outcomes of this study extend beyond the initial 3‐month intervention period to include ongoing care.

This study has some limitations. First, the findings are based on results from only one municipality; hence, it is unclear whether the same effects would be observed across other municipalities throughout Japan. Comprehensive Services for LTC Prevention and Daily Life Support allow flexible modifications according to the specific conditions of each municipality. In fact, in Taketa City, social participation destinations were prepared as follow‐up options after the short‐term intensive prevention service program. However, providing such social participation opportunities is not a mandatory requirement of this service. Therefore, it is possible that methods other than the short‐term intensive prevention service implemented in this study are used elsewhere. As of the fiscal year 2022, short‐term intensive prevention services have been implemented in 730 out of 1741 municipalities (41.9%) in Japan.^28^ Further research is needed. Second, for the non‐participant group, it was unclear what other services they might have utilized. Previous studies have shown that some participants in preventive benefit services do not experience worsening care needs compared with non‐participants.^17^ This may have led to an underestimation of the results reported in the present study.

In conclusion, this study monitored the participants and non‐participants of a short‐term intensive prevention service in Japan over a 3‐year period and compared their LTC costs, finding that participants incurred approximately 500 000 JPY less in care costs per person. Short‐term intensive prevention services contribute to cost optimization in Japan. Therefore, promoting the concept of reablement is crucial to support the sustainability of the LTCI system.

## Author contributions

Conception: RW, MS, KI, and KK; design: RW, MS, KI, and KK; analysis: RW; interpretation of the data: RW, MS, KI, and KK; writing the article: RW; data collection: RW, MS, and KK; critical revision of the article: MS, KI, and KK. All authors have read and approved the final version of the manuscript and agree with the order of presentation of the authors.

## Funding information

This study was funded and commissioned by Omron Corporation and supported by the Japan Society for the Promotion of Science (JSPS) KAKENHI (22K17409, 23H00060, 25K00727). The data of non‐participants in this study were from the Japan Gerontological Evaluation Study, which was supported by Grants‐in‐Aid for Scientific Research (20H00557, 20K10540, 21H03196, 21K17302, 22H00934, 22H03299, 22K04450, 22K13558, 22K17409, 23H00449, 23H03117, 23K21500, from JSPS), Health and Labour Sciences Research Grants from the Ministry of Health, Labour and Welfare, Japan (19FA1012, 19FA2001, 21FA1012, 22FA2001, 22FA1010, 22FG2001), Research Institute of Science and Technology for Society (RISTEX), grant number JPMJOP1831, from the Japan Science and Technology Agency (JST), a grant from the Japan Health Promotion and Fitness Foundation, a TMDU priority research areas grant, and the National Research Institute for Earth Science and Disaster Resilience. The views and opinions expressed in this article are those of the authors and do not necessarily reflect the official policy or position of the respective funding organizations.

## Disclosure statement

This study was conducted by the Japan Agency for Gerontological Evaluation Study and the Center for Well‐being and Society, Nihon Fukushi University, with funding and commission from the Omron Corporation.

## Ethics statement

Ethical approval was obtained from Nihon Fukushi University (23‐028‐02), Chiba University (M10460), and the Japan Agency for Gerontological Evaluation Study (2019‐01).

## Patient consent statement

The purposes of the use of the information were disclosed in advance, and participants were given the opportunity to opt out of the study. For non‐participants, the self‐administered questionnaire was accompanied by a description of the study, and the return of the completed questionnaire was regarded as the participant's provision of informed consent. All methods were carried out in accordance with the Declaration of Helsinki.

## Supporting information


**Figure S1.** Follow‐up periods for participants and non‐participants in short‐term intensive prevention service.

**Table S1.** Difference in service participants and cumulative cost of LTCI (stratified linear regression analysis).
**Table S2.** Association between service participants and LTCI, disability, and mortality.

## Data Availability

The datasets generated and/or analyzed during the current study are not publicly available owing to ethical considerations and research agreements, and therefore cannot be shared with third parties.
