# Supplementary material for: Difference in long‐term care cost obtained with the short‐term intensive prevention service (day service type C): A 3‐year follow‐up study of Japanese older adults
Source: Geriatr Gerontol Int. 2025 Jun 19;25(8):1058–64. doi: 10.1111/ggi.70102 (PMC12336428; doi:10.1111/ggi.70102)
Supplement: Supplementary file 1 — Figure S1. Follow‐up periods for participants and non‐participants in short‐term intensive prevention service. [file GGI-25-1058-s002.pptx]

## Slide 1
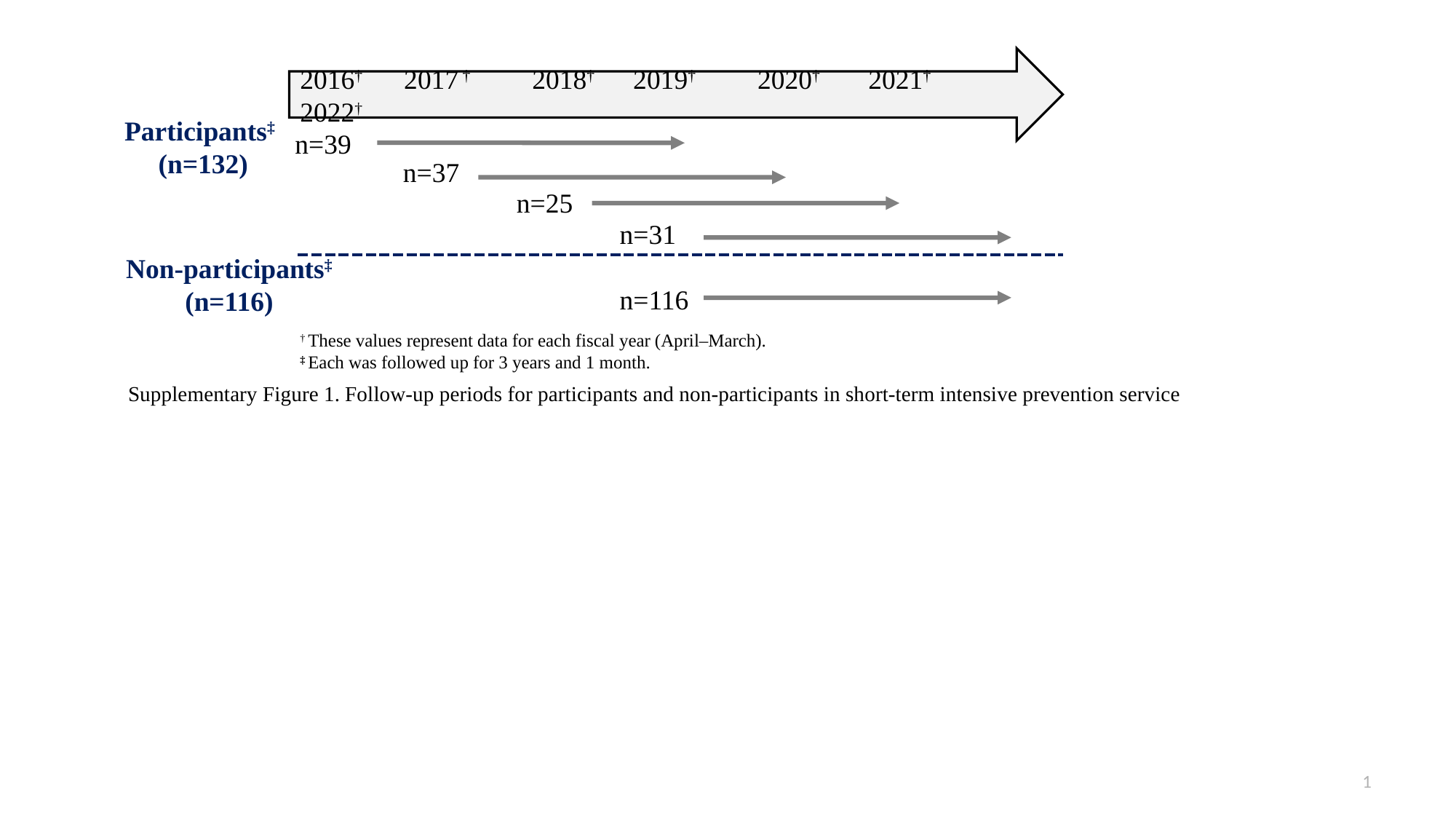

2016† 2017 †　 2018† 2019†　 2020† 2021†　 2022†
Participants‡
(n=132)
n=39
n=37
n=25
n=31
n=116
Non-participants‡
(n=116)
† These values represent data for each fiscal year (April–March).
‡ Each was followed up for 3 years and 1 month.
Supplementary Figure 1. Follow-up periods for participants and non-participants in short-term intensive prevention service
1
